# Supplementary material for: Association between waist circumference and sleep disorder in the elderly: Based on the NHANES 2005–2018
Source: PLoS One. 2024 Sep 23;19(9):e0308860. doi: 10.1371/journal.pone.0308860 (PMC11419370; doi:10.1371/journal.pone.0308860)
Supplement: Attachment — Submitted filename: Comments to the authors.docx [file pone.0308860.s001.docx]

Comments to the authors:

This study addresses an important health problem in the elderly. The study evaluates whether waist circumference (WC) is related to sleep disorder. The authors report a significant association between WC and sleep disorder particularly in higher quartiles of WC. The authors concluded that people above 60 years with larger WC were more likely to have a sleep disorder. Whilst this study is valuable, the below weaknesses need to be addressed to strengthen it.

Methods

Study population: Please provide a summary of the sample design NHANES uses for its data collection and what makes it nationally representative.

Lines 15 – 19: What was the difference in the characteristics of those with missing information and those without missing information? Address how the missing information of nearly 80% of the eligible participants above 60 years could influence your results? Will the results change if you imputed?

Line 21 – 22: Waist circumference couldn’t be a covariate but the main independent variable. It would be important to clarify that by having a separate heading for waist circumference and be clear it is the independent variable. Also provide information on the units of measurement and if any recategorization was done and why

Line 23 – 30; 1- 12: More information on how some covariates were measured but nothing was provided on covariates such as age, race, family income, recreational physical activity. Overall, this section needs some improvement including stating how the covariates were measured and categorized/recategorized.

Outcome:

How was the outcome treated? Yes or No? It seems there was information on the types of sleep disorders. Any plans for subgroup analyses with the different sleep disorders?

Lines: 11 – 12: Is the complication index a standardized index? Please provide reference if it was previously standardized. Otherwise, address the issues of standardization and validity for this index.

Statistical analysis:

Line 22: What was the basis for showing continuous variables as medians and IQRs?

Line 26: Why did you choose four categories for the WC? Any precedence for this approach?

Line 27 – 30: How did you select the confounders? How many variables were adjusted in the final model?

Line 1 – 2: How many age categories did you create for the subgroup analysis? How many racial groups did you use for subgroup analysis? And how many were collected? Any information on ethnicities?

The language in the analysis needs improvement.

Results:

Line 18: comorbidity index? Or complication index? You will need to provide more details in the methods section.

Lines 21 – 24: It is a better approach to present percentages than absolute numbers.

Association of WC with sleep disorder

Lines 17 – 21: There is lack of clarity on the sensitivity analysis. What were you testing for? Was there any suspected residual confounding by any unmeasured variable you used for the sensitivity analysis? P for trend is not a measure of sensitivity testing and not sure why that was mentioned here.

Subgroup analyses:

Lines 24 – 29: There was correlation analysis conducted here and so you may need to be mindful of the use of this term. Was there any evidence of effect modification by age, gender and race? You will need to present the results of the subgroup analyses other than the trend. Any evidence that the association differed by age, gender or race?

Discussion

Line 6 – 7: No correlation analysis was conducted, and the authors need to limit themselves to the correct terminology.

Lines 25 – 27: This is pointing to evidence of reverse causality. NHANES is a cross-sectional study and hence reverse causality is a big issue. How did you address this?

Table 3: No need to present both the betas and odds ratios information.

Table 4: Same comment as Table 4. Please present only the odds ratios. Also check the formatting of the Table
