# Supplementary material for: Association between waist circumference and sleep disorder in the elderly: Based on the NHANES 2005–2018
Source: PLoS One. 2024 Sep 23;19(9):e0308860. doi: 10.1371/journal.pone.0308860 (PMC11419370; doi:10.1371/journal.pone.0308860)
Supplement: Attachment — Submitted filename: Response to Reviewers.docx [file pone.0308860.s003.docx]

Dear editor，
Thank you for your letter suggesting revisions to our manuscript entitled, “Association between Waist Circumference and Sleep Disorder in the Elderly: Based on the NHANES 2005–2018”. We are very grateful to you and the reviewers’ comments on our manuscript and carefully considered every comment, and made revisions accordingly. Based on your suggestions, we have answered the questions one by one. Thank you for your time. All the best to you.

Most sincerely,
July 23th, 2024

RESPOND TO REVIEWERS:

**Reviewer 1**

Comment 1: For the issue of paper weighting, this is the requirement for the use of data on the NHANES website, and there are clear guidelines on how to weight the data.

Reply 1: Thank you for your professional review. We have appropriately weighted the NHANES data in our analysis in accordance with the guidelines provided on the NHANES website. Specific details on the weighting methodology used are included in the manuscript to ensure clarity and transparency. We have applied the recommended weights (e.g., WTDRD1/7) to account for the complex sampling design, which includes stratified, cluster, and multistage sampling with unequal probabilities. This approach ensures that our statistical analyses, including standard error and confidence interval estimates, are accurate and reliable.

Comment 2: Other questions I don't think the author did a good job of adjusting and revising my questions.

Reply 2: Thank you for your professional review. We appreciate your thorough review and the insights you have provided. We have done our best to make changes in accordance with your valuable comments, however, there are some limitations that cannot be well addressed. We hope to design more scientific experimental schemes in the future to further improve the scientificity of the research. Please kindly understand.

**Additional Editor**

Additional Editor Comments:

Many of the critical comments pointed out by reviewer 1 is either not addressed or the justification is not acceptable. I understand that there are some limitations but these limitations should be addressed in a scientific way or acknowledged in the limitation section. It is possible that some studies have used unweighted NHANES data but this is not a justification, you should clarify why weighing the data was not needed for your data.

Reply: Thank you. We have weighted and analyzed our nhanes data, making extensive revisions in the manuscript
